# Participants’ experiences of a counsellor-supported PTSD Coach intervention in a resource-constrained setting

**DOI:** 10.1017/gmh.2024.34

**Published:** 2024-03-11

**Authors:** Erine Bröcker, Freda Scheffler, Sharain Suliman, Miranda Olff, Soraya Seedat

**Affiliations:** 1 Department of Psychiatry, Faculty of Medicine and Health Sciences, Stellenbosch University, Cape Town, South Africa; 2 Neuroscience Institute, University of Cape Town, Cape Town, South Africa; 3 Department of Psychiatry, Academic Medical Centre, University of Amsterdam, Amsterdam, Netherlands

**Keywords:** counsellor support, internet- and mobile-based interventions, PTSD, PTSD Coach, resource-constrained setting

## Abstract

We explored participants’ experiences of a counsellor-supported PTSD Coach mobile application intervention (PTSD Coach-CS) in a randomised controlled trial. PTSD Coach-CS participants, who received the intervention and self-completed a custom-designed questionnaire at intervention completion were included (*n* = 25; female = 20; ages 19–59; isiXhosa = 22). This questionnaire comprised questions regarding the feasibility, acceptability and potential impact of the PTSD Coach-CS intervention, and general psychological support in our setting. Data were analysed using Braun and Clarke’s thematic analysis. Three main themes emerged. (i) Participants’ largely positive experiences of treatment procedures included the safe space created by the counsellor support in combination with the PTSD Coach application, allowing them to learn about and understand their lived experiences, and to accept their PTSD diagnoses. (ii) Positive perceptions of the PTSD Coach application, yet raising important concerns (e.g., lack of family involvement) for future consideration. (iii) Intervention-specific and systemic treatment barriers (e.g., stigma) providing important information to inform and increase the usefulness of the PTSD Coach-CS intervention. The findings suggest that the PTSD Coach-CS intervention may help address the need for access to suitable care for South African adults with PTSD. Some contextual barriers must be considered in further intervention implementation.

## Impact statement

Low- and middle-income countries (LMICs), such as South Africa, often face healthcare resource constraints, with many individuals not accessing the needed support. To identify feasible and effective intervention alternatives for trauma survivors in LMICs, we evaluated a counsellor-supported PTSD Coach mobile application (PTSD Coach-CS) intervention in a randomised controlled trial (RCT) in a South African adult community sample. As part of the evaluation, we explored participants’ experiences of the intervention to inform and complement the findings of the RCT. Participants experienced the intervention procedures positively, especially the counsellor’s support, who practically and effectively introduced them to the application (app), increasing app engagement. Participants experienced a safe space that, together with the app, facilitated self-acceptance of their lived experiences (e.g., trauma and PTSD). The PTSD Coach app was experienced as easy to navigate, and helpful in learning about and managing PTSD symptoms. Participants gained hope for the future and wanted others to benefit similarly. Participants provided valuable insight into the barriers (specific to the intervention and systemic-related) requiring improvement. This included spreading awareness of the PTSD Coach app as freely available, including the counsellor’s support in introducing the app’s features. Furthermore, taking the intervention to the communities may help in overcoming travel and associated financial barriers. Finally, evaluating the PTSD Coach family app in conjunction with a PTSD Coach-CS intervention may improve treatment outcomes. This was the first study to evaluate a counsellor-supported PTSD Coach app in a resource-constrained setting and participants’ experiences thereof. The findings proved useful in understanding how to improve the feasibility, acceptability and usefulness of the PTSD Coach-CS intervention for future use in a resource-constrained South Africa. We build on the existing evidence of internet- and mobile-based interventions for PTSD, suggesting that the PTSD Coach-CS intervention and the app itself can widen access to psychiatric care.

## Background

Low- to middle-income countries (LMICs), such as South Africa, face high levels of psychiatric conditions, including post-traumatic stress disorder (PTSD), impacting the daily functioning of those affected (Kessler et al., 2017; Singla et al., 2017; Ng et al., 2020). For instance, in sub-Saharan African countries, the PTSD prevalence is around 22% (Ng et al., 2020). Timeous and effective interventions can mitigate the debilitating effects of psychiatric conditions like PTSD (Seedat and Suliman, 2018; Bisson and Olff, 2021). However, compared to higher-income countries, LMICs typically face greater resource constraints limiting access to the appropriate care (Singla et al., 2017; Seedat and Suliman, 2018; Docrat et al., 2019). Broadly, barriers to appropriate care can be divided into (i) availability of treatments and (ii) accessibility to available treatments, which are influenced by both systemic and personal factors (Docrat et al., 2019; Knettel et al., 2019; Sander et al., 2020).

Docrat et al. (2019) conducted a large-scale national situational analysis evaluating the costs and resource constraints associated with psychiatric services in South Africa. The public health care (PHC) sector serves 84% of the South African population, with the remainder accessing services with private health insurance. The findings suggest a ratio of 0.31 psychiatrists per 100,000 PHC users. The national ratio of psychologists is slightly higher at 0.97 per 100,000. More promising but still lacking, the proportion of specialised nurses, albeit not all psychiatric nurses, was 80 per 100,000 users (Docrat et al., 2019). These ratios likely worsened since its publication, given the population increase from approximately 58.8 to 60.6 million registered South African citizens (South African Department of Statistics (Stats SA), 2022). Thus, a hugely under resourced infrastructure hinders the feasibility and sufficient availability of needed psychiatric care in the South African PHC setting.

Despite being under resourced, psychiatric services are available in South Africa, and it is important to recognise the substantial progress made since the inception of the Mental Health Care Act 17 of 2002 and the subsequent South African National Mental Health Policy Framework and Strategic Plan 2013–2020 (The Department of Health, 2013; Petersen et al., 2016). In South Africa, barriers to accessing available PHC services include transport difficulties, work responsibilities and, pertaining more to psychiatric services, the stigma often associated with diagnosing and treating psychiatric conditions (Knettel et al., 2019). Most PHC users walk or use public transport to reach their destinations (e.g., workplace or hospital) (Stats SA, 2021). Globally and locally, recent exorbitant fuel costs and resultant increases in the cost of living (including public transport fares) hamper the ability to use public transport (Stats SA, 2021). Coupled with a high unemployment rate, the impact of rolling load shedding (i.e., rolling electricity blackouts), and rife and often violent public transport protests, many are unable to access PHC services (Goldberg, 2015; Laher et al., 2019; Mmakwena, 2022). Finally, the harmful effects of stigma often prevent treatment-seeking individuals from accessing services (Knettel et al., 2019; Booysen et al., 2021; Monnapula-Mazabane and Petersen, 2021). This highlights the importance of identifying alternative or supplemental treatment resources and understanding how they may assist in overcoming some of the mentioned barriers.

Overall, individuals seem to have limited access to essential services and treatments in the South African context. To improve access to appropriate psychiatric care in resource-constrained settings, researchers increasingly implement and evaluate suitable treatment alternatives that require less specialised resources (e.g., counsellors as opposed to psychologists) and that are less costly (Singla et al., 2017). Internet- and mobile-based interventions are some of the more accessible and low-cost alternatives that can potentially alleviate some of the known challenges to accessing psychiatric care in resource-constrained settings such as South Africa (Olff, 2015; Ruzek and Yeager, 2017; Bröcker et al., 2022, 2023). Most of South Africa has sufficient cellular coverage with particularly high active smartphone subscriptions (approximately 65 million) (Independent Communications Authority of South Africa [ICASA] 2020). Although internet- and mobile-based augmentation of resources has great potential to meet real needs, with over 300,000 mobile health applications available, concerns regarding the applications’ credibility, privacy, and overall quality must be considered (Byambasuren et al., 2018; Chandrashekar, 2018). After reviewing the content and quality of 69 PTSD-focused applications (apps), Sander et al. (2020) highlighted the freely available PTSD Coach app as a promising treatment option with positive quality ratings. The PTSD Coach app forms part of a 20 mental health-related apps suite developed by the Veterans Affairs of the United States Department of Defence and is available for both Android and iOS users (Gould et al., 2019; United States Department of Veterans Affairs, 2022). The PTSD Coach app includes trauma and PTSD psychoeducation, tools to manage and track PTSD symptoms, and support resources (visit https://www.ptsd.va.gov/appvid/mobile/ptsdcoach_app.asp for further information). Designed for self-managed use or as augmentative treatment, the PTSD Coach app has approximately one million downloads spanning 115 countries (Hoffman et al., 2011; Owen et al., 2015).

To date, evidence of the PTSD Coach app’s feasibility and effectiveness is mostly from high-income countries with comparatively fewer healthcare resource constraints and suggests future research should include a supportive component to intervention delivery to enhance engagement and effectiveness (Kuhn et al., 2018; Bröcker et al., 2023). Research in resource-constrained contexts supports the use of less specialised mental health resources (counsellors vs. clinicians) in intervention delivery to widen access to care (Spedding et al., 2015; Singla et al., 2017; De Kock and Pillay 2018; Rossouw et al., 2018).

### Study context and rationale

To address the aforementioned gap in the extant literature on PTSD Coach, we conducted a randomised controlled trial (RCT) to evaluate the effectiveness of a brief four-session counsellor-supported PTSD Coach mobile app (PTSD Coach-CS) intervention in South African adults with PTSD (trial registration number: PACTR202108755066871) (Bröcker et al., 2024). Study flyers were distributed in the community and promoted on social media. The RCT allocated participants to PTSD Coach-CS or enhanced Treatment-as-Usual (e-TAU) on a 1:1 basis ((Bröcker et al., 2024). RCT participants were aged 19–61 years; female = 89%; black = 77%, owned smartphones, could provide written informed consent, and were conversant in English. The latter was a requirement as the original version of the PTSD Coach app was used.^1^ A registered clinical psychologist conducted the Mini-International Neuropsychiatric Interview for DSM-5 and the Clinician-Administered PTSD Scale for DSM-5 (CAPS-5) to confirm their PTSD diagnosis (Sheehan et al., 1998; Weathers et al., 2018). The psychologist, blinded to intervention allocation, assessed treatment response from pre- to post-treatment at 4 weeks and to 1 and 3-month follow-up. Outcome measures included the CAPS-5 (main outcome), the PTSD Checklist and the Depression, Anxiety and Stress Scale-21 items self-report measures (Henry and Crawford, 2005; Wortmann et al., 2016). User experiences of the PTSD Coach app were also collected with self-administered surveys (Kuhn et al., 2014). RCT participants were not financially compensated but were reimbursed for their transport costs (ZAR200/USD13.30)^2^ at each study visit.

The main findings of the RCT included greater improvement in clinician-monitored PTSD and self-reported stress symptoms over time in the PTSD Coach-CS group compared to the e-TAU group (Bröcker et al., 2024). The intervention uptake was good, with most participants attending all four intervention sessions. Also, self-reported app use outside of these sessions varied from daily to five times per week (Bröcker et al., 2024). Generally, the PTSD Coach app was positively received, and participants rated the app as moderately to very helpful in managing PTSD symptoms. Smartphone ownership was not a significant barrier to intervention implementation, but technical difficulties related to app download were problematic for one participant (Bröcker et al., 2024). Overall, the RCT results supported the feasibility and acceptability of both the original version of the PTSD Coach mobile app platform and the notion of involving counsellors in intervention delivery (Bröcker et al., 2024). For more information on the original RCT (e.g., counsellor training and protocol fidelity, please see Bröcker et al., 2024).

### Present study aim

One of the secondary objectives of the RCT was a qualitative sub-study to augment the findings of the RCT (Bröcker et al., 2024). To this end, we collected qualitative feedback from PTSD Coach-CS participants exploring their experiences of the feasibility (e.g., how they experienced the support received), acceptability (e.g., what they liked about the intervention) and impact (e.g., how the intervention influenced them) of PTSD Coach-CS in a South African community setting. Qualitative data provide a more in-depth understanding of participants’ experiences, which can be used to inform future research and intervention implementation. This manuscript presents the results of the qualitative sub-study.

## Methods

We adhered to the COnsolidated criteria for REporting Qualitative research guidelines when writing this manuscript (Tong et al., 2007).

### Participants

Of the 32 participants allocated to the PTSD Coach-CS intervention, four were lost to follow-up at post-treatment, while three participants indicated that they did not have time to complete the qualitative sub-study as they had to return to work. Thus, 25 PTSD Coach-CS intervention participants completed the 12-item questionnaire (see ‘Data collection’ for a more detailed description). Participants in this qualitative sub-study (aged 19–59 years; mean = 38.9; SD = 12.7) were predominantly female (80%), black (77%) and isiXhosa speaking (80%) (see Table 1 for additional socio-demographic information).

**Table 1.** Baseline socio-demographic characteristics

Variable	*n* (%)
Sex	
Female	20 (80)
Male	5 (20)
Ethnicity	
Black	22 (88)
Mixed–race	2 (8)
White	1 (4)
First language^3^	
Xhosa	20 (80)
Afrikaans	4 (16)
English	1 (4)
Highest level of education	
Secondary	
Grade 9	2 (6)
Grade 10	2 (6)
Grade 11	6 (19)
Grade 12	15 (47)
Tertiary	7 (22)
Marital status	
Single	18 (56)
Married/living together	11 (34)
Divorced/separated	2 (6)
Widowed	1 (4)
Annual income level^1^	
<ZAR 10000 (USD 540)	19 (76)
ZAR 20–40,000 (USD 530–1,060)	3 (12)
ZAR 40–60,000 (USD 1060–2,120)	1 (4)
ZAR 60–100,000 (USD 2120–3,180)	1 (4)
>ZAR 100 000 (USD 3180)	1 (4)

### Intervention

The intervention entailed four weekly in-person counsellor-supported sessions lasting approximately 30–40 min each. These sessions were conducted in English, aligning with the study eligibility criteria that required participants to be conversant in English. However, as stated in the study informed consent, participants were reminded that they could request assistance (e.g., support in their first language) if needed. At each session, the registered counsellor went through and discussed selected tools of the app (see Table 2). The counsellor provided a standard method of support (e.g., assisting with language and technology difficulties without providing therapeutic input) as per the intervention protocol. During session 1, the counsellor assisted in installing the PTSD Coach app on participants’ phones, with mobile data provided by the study. All four sessions followed the same structure: (i) setting an agenda for the session; (ii) gathering feedback on the last session; (iii) reviewing the week and homework; (iv) working through selected symptoms and tools with the counsellor to assist with technical difficulties and (v) agreeing on homework for the following week. The app addresses eight PTSD-related symptoms with accompanying 23 tools and activities. Tools for each session were selected after carefully considering their suitability and appropriateness to our setting. Participants were encouraged to use the app outside the intervention sessions.

**Table 2.** Intervention content

Session	Dashboard	Tools	Activity
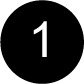	Learn	About PTSD	What is PTSD?
		Getting professional help	How does PTSD develop? Is counselling confidential?
		PTSD and the family	Fighting fair
	Manage symptoms	Reminded of the trauma	Body scan with Julia Deep breathing
		Avoiding triggers	RID: Coping with triggers Observe thoughts
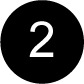	Manage symptoms	Disconnected from people	Connect with others Relationship tools
		Disconnected from reality	Grounding Muscle relaxation
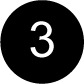	Manage symptoms	Sad/hopeless	Seeing my strength Change your perspective
		Worried/anxious	Mindful breathing Thought shifting
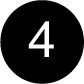	Manage symptoms	Angry	Mindfulness Positive imagery
		Unable to sleep	Good sleep habits Ambient sounds

### Data collection

Directly after completing intervention session 4, the counsellor invited PTSD Coach-CS participants to complete the 12-item questionnaire (paper-and-pen).^4^ This self-administered questionnaire elicited data on the feasibility, acceptability and the potential impact of the PTSD Coach-CS intervention (see Supplementary material for further details). Questions also covered the feasibility and acceptability of psychological support in our setting in general, to further inform intervention adaptation and the effects of the COVID-19 pandemic on app use. An open-ended item concluded the questionnaire to collect any additional relevant information. A research assistant anonymised, captured and grouped the responses per question in a Microsoft Excel spreadsheet. The semi-structured and open-ended nature of the questions leant themselves to qualitative analysis.

### Data analysis

Two researchers (E.B. and F.S.) with qualitative research experience, independently conducted thematic analysis (Braun and Clarke, 2006), using Atlas.ti (Version 23.0.8.0) (ATLAS.ti, 2022). E.B. is a registered clinical psychologist and F.S. is a registered research psychologist. We followed the six steps proposed by Braun and Clarke (2006): (i) reading and re-reading through the transcripts to familiarise ourselves with the data and obtain a general understanding of participants’ responses; (ii) generating initial codes line by line from the transcripts until reaching saturation (see Supplementary material for further details); (iii) constructing sub-themes from the codes; (iv) reviewing and collapsing the potential sub-themes; (v) defining and naming the main themes and (vi) synthesising the themes into a coherent whole (i.e., writing the manuscript). Considering the shared meaning-making inherently involved in qualitative analysis, the second coder (F.S.) was not involved in the larger study and conducted an independent analysis. Discrepancies in codes, themes and sub-themes were resolved through discussion.

### Ethical considerations

The main study (RCT) was approved by the Health Research Ethics Committee (HREC) of SU (N18/03/061/ S18/05/058). During the informed consent process of the RCT, participants were informed of the voluntary nature of all study procedures (including the qualitative sub-study). All trial data were anonymised to protect participants’ privacy and ensure confidentiality.

## Results

**Figure 1.**
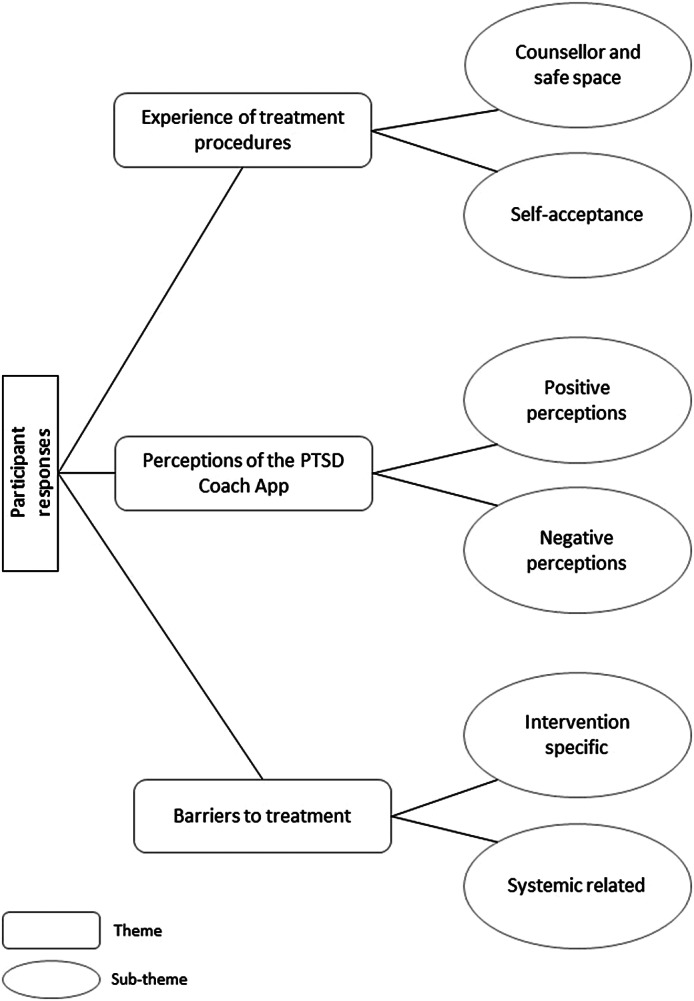
Thematic map demonstrating themes and sub-themes.

Three main themes emerged from participants’ responses: (i) experience of treatment procedures, (ii) perceptions of the PTSD Coach app and (iii) barriers to treatment. The themes and sub-themes are presented in Figure 1 and discussed with erbatim quotations supporting our findings.

### Theme 1: Experience of treatment procedures

Most participants were positive towards the intervention procedures. The counsellor support created a safe space for the participants to talk and ask questions, which, in combination with the PTSD-Coach app tools (see Theme 2), allowed them to experience self-acceptance and understanding of their PTSD diagnosis.

#### Counsellor support and safe space

Participants found the intervention procedures allowed them to talk freely about their experiences: “*I like that I was able to speak about what was bothering me*” (P17). Participant 5 said: “*…I can talk about the things….”* Since the PTSD Coach-CS intervention protocol instructed the counsellor to maintain strict boundaries and not provide therapy, participants likely referred to the structured elements (e.g., gathering feedback on the previous session) and overall counsellor involvement that was perceived as therapeutic. It is also possible that the participants referred to the monitoring sessions with the psychologist.

Participants’ overall experience with the counsellor was positive. For example, participants said: *“Better than good. She was so patient very good”* (P2) and *“She was very helpful and supportive towards me. She made sure I was welcomed, and she explained everything to me”* (P20). The counsellor support was also helpful, specifically related to the app “…*because of the way things are explained in details* [sic] *for you to understand”* (P8). Furthermore, the counsellor was integral in creating a safe space that allowed them to talk freely, without judgement. For example, Participant 7 said: *“Feels* [sic] *that I came into a space where I can just be myself and learn”*, while Participant 14 said: *“It was very good she was understanding and nice, I felt free around her…I liked the fact that I had someone to talk to without a fear of being judged.”* Here, Participant 14 also alluded to fearing judgement (which can be linked to stigma) as a barrier to accessing support resources (see Theme 3).

#### Self-acceptance

The perception and experience of the counsellor support sessions as a safe space where participants felt free to talk and ask questions, in combination with the PTSD Coach app tools (see Theme 2), facilitated participants’ self-acceptance and understanding of their PTSD diagnosis. Participants said that the process *“…allows me to know that it is wise to forgive myself”* (P4) and *“It also helped me change the view I have about myself”* (P9). Similarly, Participant 22 said: *“Made me realise or shall I say find myself and see things different than before in a better way.”* Notably, Participant 20 said: *“…it made me realised* [sic] *that having PTSD is not the end of the world.”* The latter links to the barrier of denial (Theme 3). Relatedly, Participant 17 later noted that people do not seek treatment due to *“Not being able to accept that they suffer from PTSD.”*

### Theme 2: Perceptions of the PTSD Coach app

Overall, participants had a positive perception of the PTSD Coach app, with a significant emphasis on learning about and managing their PTSD symptoms, which provided hope for the future. Participants also wanted others to know about the intervention and to benefit similarly. However, some participants noted negative aspects of the PTSD Coach App.

#### Positive perception of the PTSD Coach app

Most participants found that *“PTSD Coach is a very good app and its* [sic] *very helpful”* (P18). Participants also found that *“…it was an easy app to understand”* (P13) which they could *“…use it for life”* (P24). There were specific app tools that they found helpful, such as the ‘*Inspiring quotes*’ tool that *“…motivated me* (and meditating) *about my feelings through video”* (P23), and *“*(t)*he exercise that when you feel bad you put headphones on and walk, the connection with mother nature”* (P4). Participant 4 likely referred to the ‘Leisure activities’ tool, which together with ‘Inspiring quotes’, were not part of the intervention protocol (see Table 2). This suggests that participants engaged with the app outside the counsellor-supported sessions.

##### Learning about PTSD and managing symptoms

Participants found the app’s functions of learning about PTSD and managing their symptoms helpful. This was facilitated through the “[e]*explanations about PTSD* [which] *is clear*” (P23) and links to the positive perception of the PTSD Coach app as “…*an easy app to understand*” (P13). Participants mentioned that learning about PTSD helped to “[understand] *my symptoms*…" (P12) and the app *“…help*[s] *people learning and understanding what they are experiencing”* (P21). Learning about and understanding their experiences likely enabled participants to apply the tools to manage their symptoms as the app *“…helped* [me] *to manage my stress from the trauma”* (P16). Also, Participant 9 said: *“I liked knowing where all the emotions come from and also that I know how to cope and handle those emotions for the first time.”* Participants specifically noted that *“I don’t feel angry anymore… and I know how to manage my temper…”* (P20).

##### Hope for the future

A valuable impact of the intervention is that participants experienced hope for the future. For example, participants said the intervention made “…*me realise there is still hope*” (P1) and “…*gave me the courage to go out there”* (P8). Participant 9 made the following striking comment:

*Before I came here, I thought that there isnt* [sic] *a reason for me to live and since coming here I feel stress free and I have hope for my life. I can now also handle things coming my way.*

##### Want others to benefit

Given the positive feedback about the counsellor and the app, it was unsurprising that most wanted others to benefit from it. Specifically, Participant 1 said that they *“*[w]*ant to share* [it] *with other people,”* and Participant 8 said: “…*that even myself* [I’m] *thinking of helping others the same way I was helped.”* Participant 23 who said: *“I wish all of us can be reached or having* [sic] *it in their phones,”* also alluded to the possible barrier of treatment (see Theme 3) related to unawareness of the freely available PTSD Coach app as a resource.

#### Negative perceptions of the PTSD-Coach app

Participant 3 did not find the app helpful, stating: *“In my experience the app did not help* (instead) *the people encouraged me to talk about my feelings and that it is okay,”* implying that the counsellor support or possibly the monitoring sessions with the psychologist was of greater help than the app. Some participants were positive towards the app, but they also mentioned that they “…*would have wanted more counselling support”* (P5) and recommend to *“Ask more about emotions during sessions”* (P5). While this further highlights the importance of the counsellor-supported aspect of the intervention process, it also implies that fidelity to the RCT intervention protocol was maintained (i.e., no therapeutic input was provided).

Participants also highlighted unhelpful aspects of the app that can be improved on such as “…*Unhelpful – that it doesn’t involve the whole family"* (P17). Participant 23 mentioned that even though the app helped her to become aware that she struggles with PTSD, she found it *“…exhausting with the exercises.”* However, one should bear in mind that the intervention protocol involved working through the tools in a specific timeframe, whereas self-guided use of the app does not. Further, Participant 8 said she disliked that “*…it does ask me a lot about my experiences of trauma."* While the app itself does not require this, it is likely that Participant 8 referred to the monitoring sessions with the psychologist. Participant 8’s comment also alluded to fearing stigma as one of the barriers to accessing support resources (see Theme 3).

### Theme 3: Barriers to treatment

Most participants found the PTSD Coach-CS intervention positive and the app itself beneficial, but there were some barriers to its implementation. These were both specific to the current intervention process and related to systemic barriers to accessing support in our setting.

#### Barriers to engaging with the PTSD Coach-CS intervention

Two participants noted that the distance they travelled to attend the sessions was problematic. Likewise, travelling concerns were mentioned as a systemic barrier to accessing support.

Participants indicated that people are unaware of the PTSD Coach app as a freely available resource and noted a need for the app to be explained first. For example, while Participant 23 said *“*…*everyone should have it on their phones…,”* they continued, *”… in hospitals someone to help them using the app."* Accordingly, Participant 17 said: *“Being introduced to the app that I can also use at home was very helpful.”* Additionally, participants highlighted that the intervention could be beneficial *“By attending sessions”* (P12) and *“By listening* [to] *what it said and practice it”* (P15).

Concerning the recent COVID-19 pandemic and the impact thereof on accessing support, it was promising that participants’ use of the PTSD Coach app was mostly unaffected by the pandemic at the time: *“It has no impact to* [sic] *me because I am able to open my app anytime and anywhere”* (P4). While two participants noted a negative impact, it was not clear if they meant regarding the use of the app (i.e., having less time to use the app as they had to look for new job opportunities) or regarding a general deleterious effect of the pandemic ("*Negatively impact regarding job seeking*“ (P11); and ”… *negatively because some people lost their jobs and their loved ones*“ (P22), respectively).

#### Systemic barriers to accessing treatment

Barriers to accessing treatment included travel distances and associated costs, unawareness of needing help and existing resources, stigma associated with accessing psychiatric support and language barriers.

##### Travelling

Participants said: *”Travelling takes too long to travel with taxis"* (P1) and *“…many people do not have travel money”* (P2). While referring to intervention procedures, Participant 18 said: “*The money provided is really helping because I take three taxis to attend a session.”* This highlights that our intervention procedures addressed some systemic barriers to accessing support related to transport and financial constraints. Importantly Participant 19 said that *”…others are afraid of traveling* [sic] *every time",* possibly referring to violent taxi protests or general crime as an added barrier in our setting.

##### Unawareness

Unawareness of existing resources was reported as a barrier: *“…lack of information, some people do not know where to go in order to get the treatment”* (P16); *“We don’t know where to seek help ….”* (P20); and *“…* [people] *are not aware that there is treatment for it and that they can get help”* (P18).

Additionally, unawareness of needing help, or denial, was mentioned as a barrier for people to accessing support: *“Some people don’t see that they need help until something happens …”* (P3), and *“They are in denial or they do not want to accept their situation or scared of what people will say”* (P14).

##### Stigma

Participant 14’s statement that they are “*… scared of what people will say…"* signals the barrier of fear of accessing existing support. Participants said that *“*[t]*hey are afraid to get help because they feel they are going to be judged”* (P5). Participant 12 noted culture-related stigma as a barrier: *“They think you’re insane or too western,”* suggesting cultural disapproval of accessing support. Participant 7 mentioned gender-related stigma since in *“*[t]*he past it was just for women and children … men were seen as weak when they seeked* [sic] *support.”*

General “*stigma*” (P3) about accessing mental health support was reported by participants: “*I think people that ’bully’ a person with PTSD are barriers”* (P13). Most participants heard about and became involved in the intervention through word-of-mouth, indicating that peer referral and support may decrease fear and stigma and increase social acceptability of receiving help. Further, this fear and acceptability of receiving help was demonstrated by Participant 14 who said: *“I can now see there is no problem in seeking help.”*

##### Support

Participants’ responses indicated a perceived lack of resources as contributing to treatment barriers, noting *“Availability of counsellors,”* (P4) and that “*The clinics in SA does not provide the support people want – Therefore people have nowhere to go”* (P11).

Conversely, some participants mentioned an unwillingness to engage with existing resources as a factor that prohibits people from receiving the care they need. This unwillingness was multi-layered as some of it was related to counselling specifically as *“People don’t take counselling serious”* (P16) and *“They think it’s a waste of time…”* (P21). More general comments were that “*…they don’t want to tell strangers their problems”* (P17) and *“Others they decide not to seek help…”* (P22). These factors likely connect with previously discussed themes as a lack of awareness of what available services constitute and stigma could be drivers of these perceptions. There was also the perception that accessing support will *“Remind them of their trauma”* (P6). One participant mentioned language as a barrier, saying that while some people do not know how to access care, *“others can’t speak English…”* (P21).

## Discussion

This qualitative study explored participants’ experiences of a PTSD Coach-CS intervention for adults with PTSD in a South African resource-constrained setting. The emergent themes related to (i) experience of treatment procedures, (ii) perceptions of the PTSD Coach app and (iii) barriers to treatment. Overall, participants experienced both the counsellor support and the PTSD Coach app as positive. The findings indicated that participants perceived the human interaction component of the treatment procedures as particularly positive and beneficial. While the counsellor and psychologist’s involvement in the intervention and monitoring sessions were not meant as therapeutic, participants seemed to have experienced it as such (Brown et al., 2014; Christopher et al., 2017; Thong et al., 2016). This links to participants’ concerns regarding the lack of human interaction mentioned during a study evaluating experiences with a PTSD Coach Online programme (using a web-based platform) intervention (Ellis et al., 2022). Thus, our findings further support the value of a blended (i.e., human component and mobile-based) approach noted in PTSD Coach-focused research (Owen et al., 2015; Cernvall et al., 2018; Shakespeare-Finch et al., 2020).

The positive perception of the PTSD Coach app in our setting is encouraging. Similar to prior PTSD Coach-focused research, our participants found the app’s functions concerning learning about and managing PTSD symptoms helpful (Miner et al., 2016; Cernvall et al., 2018; van der Meer et al., 2020). Learning about PTSD refers to psychoeducation, which plays a vital role in treatment and often forms the foundation for more specialised intervention strategies (Whitworth, 2016; Taylor, 2022). Previous PTSD Coach-focused research commented on participants’ increased openness for further support after learning about and understanding their symptoms (Kuhn et al., 2014; Ellis et al., 2022). Participants’ negative perceptions of the app align with dissatisfaction with the app, noted in prior PTSD Coach app research (i.e., perceived need thereof) (Pacella-LaBarbara et al., 2020; Hensler et al., 2022;). The lack of family involvement mentioned reiterates that PTSD affects both the family and the trauma-exposed individual. To this end, the PTSD Family Coach app was developed to assist families of those affected by PTSD (Gould et al., 2019). Participants’ dislike of detailed enquiry about the experiences of trauma was likely in reference to the monitoring sessions with the psychologist rather than the intervention or the app itself (Bröcker et al., 2024). This potential participant discomfort during PTSD-focused research is reported in the literature with caution requested when conducting such research (Legerski and Bunnell, 2010; Brown et al., 2014). However, it could also be that participants were alluding to the stigma associated with accessing psychiatric support and a lack of knowledge or misconception of what treatment entails (Morgan et al., 2018; Knettel et al., 2019; Booysen et al., 2021).

Other negative comments were largely related to the desire for more human interaction and more counselling. This highlights participants’ desire for direct assistance/therapy that they may not be receiving due to resource constraints (Docrat et al., 2019). The counsellor support in combination with the PTSD Coach app created a safe space for participants to learn about and understand their lived experiences and to accept their PTSD diagnoses. Mental illness and associated diagnoses can elicit varied reactions with equally varied consequences (Hundt et al., 2019). While it can result in a denial of diagnosis and prevent treatment seeking and accessing appropriate support, it appears that for many participants, it initiated a healing process and post-traumatic growth (PTG) (Hundt et al., 2019; El-Gabalawy et al., 2021). The renewed hope for the future and wanting others to benefit similarly is associated with PTG.

The importance of hope in the context of adversity, including mental and physical illness, is central to supporting individuals facing such adversities (Long, 2018). Diminished hope tends to drive suicidal ideation, while fostering a sense of hope is often pivotal in managing mental illness such as PTSD (Long, 2018; Koenig et al., 2020). The renewed sense of hope resulted in participants experiencing the ability to see new possibilities and a feeling of self-empowerment (El-Gabalawy et al., 2021; Levi et al., 2012). Furthermore, participants wanting others to benefit similarly speaks to a sense of altruism and prosocial behaviour, which also forms part of PTG (El-Gabalawy et al., 2021). This notion of wanting others to benefit was noted in the first study to evaluate the PTSD Coach app with participants recommending the app to friends and family (Kuhn et al., 2014). Consideration of the intervention-specific and systemic treatment barriers is important to inform and increase the usefulness of the PTSD Coach-CS intervention in our setting. Participants suggested both an unawareness of the freely available app and the need for an introduction to the app as barriers to intervention engagement. This emphasises earlier discussion on the value of augmenting internet- and mobile-based interventions with guidance (i.e., human interaction) to increase both awareness of the app and engagement (Owen et al., 2015; Cernvall et al., 2018; Shakespeare-Finch et al., 2020). The described unawareness of the app extends to the systemic barrier of unawareness of existing resources. As mentioned, while the South African PHC system is overburdened, services are available and substantial progress has been made since implementing the South African National Mental Health Policy Framework and Strategic Plan 2013–2020 (The Department of Health, 2013). Lack of information on available services and difficulty navigating a referral or treatment pathway is a known global barrier to accessing support (Newman et al., 2015; Muhorakeye and Biracyaza, 2021). This highlights the importance of creating awareness of available services (i.e., clinics) and the clinics remaining informed of relevant intervention studies where individuals can receive support and alleviate overburdened PHC services. Continuous fostering of a good relationship between academia (e.g., research studies’ sites) and practitioners on the ground is mutually beneficial (Rynes et al., 2017). The barrier of travelling and associated costs is common in resource-constrained settings and is well documented in South Africa as many people use public transport or walk to their destinations (Syed et al., 2013; Schmitz et al., 2019; Stats SA, 2021). In our setting, many factors influence this barrier, such as increased public transport fees attributable to exorbitant fuel price increases and rolling blackouts, high unemployment rates, violent public transport protests or general crime (Goldberg, 2015; Laher et al., 2019; Mmakwena, 2022). Therefore, bringing psychological support to the communities can further overcome barriers to treatment (Singla et al., 2017; Rossouw et al., 2018).

Denial about needing support, and stigma (e.g., fear of judgement, and associated concerns about the social acceptability of accessing support) were mentioned as systemic barriers to treatment (Hundt et al., 2019; Ciccolo et al., 2022; Rice et al., 2020). The harmful effects of general stigma associated explicitly with mental illness are well-known (Clement et al., 2015; Knettel et al., 2019; Booysen et al., 2021; Monnapula-Mazabane and Petersen, 2021). Notably, a systematic review and meta-analysis indicated that stigma is one of the most common barriers to seeking care (Morgan et al., 2018). The stigma and fear of social rejection may be further influenced by both cultural and gender-related factors (Venter et al., 2019; Rice et al., 2020; Ciccolo et al., 2022), highlighting the need for sensitivity towards cultural and gender-based concerns when developing and promoting support resources (Ciccolo et al., 2022). A PTSD Coach Online (web-based) study describes their process of fully adapting the programme and creating PTSD Coach Online-Arabic (Miller-Graff et al., 2021; Ellis et al., 2022). Their procedures highlighted the significant role of language, gender and cultural stigma in treatment-seeking individuals in the Middle East. Our findings also indicated that language should be considered when promoting support resources, including translating intervention content into various languages, especially in a country such as South Africa. South Africa has a multicultural and multilingual population, and individuals accessing healthcare services, and healthcare providers, face language barriers. Addressing these barriers in an effort to improve linkages to care is a priority (Tate et al., 2016; Tönsing et al., 2019; Hagan et al., 2020; van Vuuren et al., 2021).

## Conclusion

This was the first study to qualitatively evaluate a counsellor-supported PTSD Coach app in a resource-constrained setting. We build on the existing evidence of internet- and mobile-based interventions for PTSD, specifically for the PTSD Coach app, and suggest that the counsellor support and the app itself are useful in our resource-constrained setting and can widen access to psychiatric care. Further, our qualitative findings are useful in understanding how to improve the feasibility, acceptability and usefulness of the PTSD Coach-CS intervention for future use. Our findings suggest that participants experienced the PTSD Coach-CS intervention as positive, especially the counsellor’s support, as they enabled a practical and effective introduction to the app and increased engagement with the app. This also created a safe space that, together with the app, facilitated participants’ self-acceptance of their lived experiences. Similarly, the PTSD Coach app itself was positively received, easy to navigate and helpful to learn about and manage their symptoms. Participants gained hope for the future and wanted others to know about the intervention and the app to benefit similarly.

Notably, the data provided insight into the barriers and what needs to be improved on in terms of intervention implementation. For instance, training registered counsellors, preferably from the respective communities, to provide the PTSD Coach-CS intervention at local clinics under supervision limits travel and associated costs. Utilising counsellors from the same communities as participants, who are more likely to be of similar culture and language as the treatment seeking individuals, can also overcome cultural and language barriers in part, as well as assist with reducing stigma associated with receiving psychiatric support. Adapting the PTSD Coach app culturally and language-wise (e.g., isiXhosa or Afrikaans) can further assist in greater intervention adoption and usefulness; however, the counsellor support might be a better use of resources and of value to PTSD sufferers.

Finally, it would be prudent to evaluate whether a PTSD Coach Family app intervention administered in conjunction with an individual PTSD Coach app intervention yields better outcomes. In summary, this study showed that the PTSD Coach-CS intervention can be a suitable intervention in a resource-constrained setting such as South Africa and provides guidance for future research and clinical use.

### Limitations

This study has several limitations. First, the nature of the data collection (i.e., paper-and-pencil questionnaires) did not allow for clarification and further exploration of responses as a verbal interview would have. Future research should use verbal interviews to allow for clarification and richer data. Relatedly, adding a usability questionnaire, such as the mHealth App Usability Questionnaire, can further enrich the data. Second, while the immediate completion of the questionnaires limited recall bias as the intervention experience was still fresh, the counsellor’s proximity may have influenced their response to be more favourable towards the counsellor. Future research should employ an independent team member to gather qualitative data. Finally, while attempts were made to contact non-completers of the RCT, none were reached. It should be noted that non-completers may have provided valuable insights into improving the app and intervention as they presumably had reasons for dropping out of the study.

In summary, this is the first study to evaluate the PTSD Coach app’s feasibility and utility in an LMIC resource-constrained setting. The findings suggest that a four-session PTSD Coach app intervention is a more accessible, low-cost alternative to treatment that can be augmented with counsellor support, which constitutes suitable care for South African adults with PTSD.

## Supporting information

Bröcker et al. supplementary material 1 Bröcker et al. supplementary material

Bröcker et al. supplementary material 2 Bröcker et al. supplementary material

Bröcker et al. supplementary material 3 Bröcker et al. supplementary material

Bröcker et al. supplementary material 4 Bröcker et al. supplementary material

## Data Availability

The data that support the findings reported in this study are not shared publicly for reasons of privacy; however, they can be made available from the corresponding author (E.B.) upon reasonable request.

## Footnotes

1 Due to cost constraints, the original version of the app was not adapted before use in the RCT. However, first, conducting an RCT and subsequent qualitative sub-study allowed us to gain insight into whether and what adaptation is needed; thereby, preventing unnecessary costs for adaptation.

2 Exchange rate in June 2023 used for all currency conversion calculations.

3 Twelve official languages of South Africa include Afrikaans, English, isiXhosa, isiZulu, isiNdebele, Sepedi, Sesotho, Setswana, siSwati, Tshivenda, Xitsonga and South African Sign Language.

4 Originally, the methodology for this qualitative sub-study included in-person individual qualitative interviews. As a more standard practice in qualitative research, this approach would have allowed for greater exploration and richer data as mentioned in the limitations of the manuscript. However, due to COVID-19 pandemic setbacks (delay in the initiation of the RCT and regulations placed in research and uncertainty at the time regarding the course and the influence of the pandemic and associated regulations on study procedures), we opted for a questionnaire in order to gather data quickly and safely.

## Supplementary material

The supplementary material for this article can be found at http://doi.org/10.1017/gmh.2024.34.
