# Supplementary material for: Participants’ experiences of a counsellor-supported PTSD Coach intervention in a resource-constrained setting
Source: Glob Ment Health (Camb). 2024 Mar 11;11:e36. doi: 10.1017/gmh.2024.34 (PMC10988172; doi:10.1017/gmh.2024.34)
Supplement: Bröcker et al. supplementary material 1 — Bröcker et al. supplementary material [file S2054425124000347sup001.pdf]

Stellenbosch
UNIVERSITY
IYUNIVESITHI
UNIVERSITEIT

Date: 26.02.2024

RE: Response to Reviewer 1

I think the authors have done a very nice job responding to these reviews, and the manuscript is much clearer. I have only two remaining (very minor) comments. First, the clarification around English/lack of translation was very useful. But it would also be helpful to clarify whether or not counselor support sessions were also conducted in English or if they were conducted in another language (e.g., the first language of the participant). Second, the table presented in the response to reviewers in response to the query about theme saturation is very useful, and I would recommend including it in the Appendix.

Thank you for the positive feedback and the raised queries that continue to improve the manuscript's quality.

In response to the query concerning the counsellor support sessions, the following was amended (Amended manuscript: p.5, Lines 158 - 160 under the heading 'Intervention.'):

> The intervention entailed four weekly in-person counsellor-supported sessions lasting approximately 30-40min each. These sessions were conducted in English aligning with the study eligibility criteria required participants to be conversant in English. However, as stated in the study informed consent, participants were reminded that they could request assistance (e.g., support in their first language) if needed.

Concerning the table about theme saturation, the amended table (indicating the number of responses analysed) is submitted as Appendix A: Questionnaire and response frequencies (please note amended table title).

*The applicable section was amended as follows (**Amended manuscript: p. 1, Lines 177 - 178, under 'Data collection'):***

> Directly after completing intervention session four, the counsellor invited PTSD Coach-CS participants to complete the 12-item questionnaire (paper-and-pen). This self-administered questionnaire elicited data on the feasibility, acceptability and the potential impact of the PTSD Coach-CS intervention (see Appendix A: Questionnaire and response frequencies for further details).

AND

(Amended manuscript:* pgs. 5- 6, *Line 191, under 'Data analyses'):

> We followed the six steps proposed by Braun and Clarke (2006): (i) reading and re-reading through the transcripts to familiarise ourselves with the data and obtain a general understanding of participants' responses; (ii) generating initial codes line by line from the transcripts until reaching saturation (see Appendix A: Questionnaire and response frequencies for further details; (iii) constructing sub-themes from the codes; (iv) reviewing and collapsing the potential sub-themes; (v) defining and naming the main themes; and (vi) synthesising the themes into a coherent whole (i.e., writing the manuscript).

E. Bröcker
