# Supplementary material for: Participants’ experiences of a counsellor-supported PTSD Coach intervention in a resource-constrained setting
Source: Glob Ment Health (Camb). 2024 Mar 11;11:e36. doi: 10.1017/gmh.2024.34 (PMC10988172; doi:10.1017/gmh.2024.34)
Supplement: Bröcker et al. supplementary material 2 — Bröcker et al. supplementary material [file S2054425124000347sup002.pdf]

Stellenbosch
UNIVERSITY
IYUNIVESITHI
UNIVERSITEIT

Date: 07.02.2024

RE: Response to Reviewers

Thank you for the detailed review and feedback. It informed the revisions and improved the quality of the manuscript. Below are my responses including the changes made which are indicated with 'tracked changes' in the submitted document titled 'Amended_Manuscript_with tracked changes'.

The primary outcomes of the randomised controlled trial have been published and forms the basis of the response to a few of the reviewer' queries and suggestions:

Bröcker E, Olff M, Suliman S, Kidd M, Greyvenstein L, and Seedat S. (2024) A counsellor-supported 'PTSD Coach' intervention versus enhanced treatment as usual in a resource-constrained setting: A randomised controlled trial. *Cambridge Prisms: Global Mental Health*, 1–25. https://doi.org/10.1017/gmh.2023.92.

REVIEWER 1

Comments to the Author

Congratulations on a well-written manuscript. Some thoughts and edits below.

The manuscript meets the requirements for a qualitative study.

1. **p.2 line 23-31 it seems a repeat of statement**

*The applicable section was amended as follows (**Amended manuscript: p. 1, Lines 22 - 31, under 'Impact Statement'):***

> Low to middle-income countries, such as South Africa, often face barriers to healthcare with many individuals not accessing needed support due to resource constraints. To identify feasible and effective intervention alternatives for trauma survivors in these countries, we evaluated a counsellor-supported PTSD Coach mobile application (PTSD Coach-CS) intervention in a randomised controlled trial in a South African adult community sample. As part of the evaluation, we explored participants' experiences of the intervention to inform and complement the findings of the RCT.

2. **p4 line 97 and line 105 - 108 is confusing, not sure what the 2 sets of data refer to. They differ? one is female 89% other female 80%**

Thank you for raising this. We amended the background to better distinguish the characteristics of participants in the RCT and those participants in the PTSD Coach-CS intervention arm who completed the questionnaire. We specifically added a section 'Study context and rationale' to better distinguish between the RCT and the qualitative sub-study described in the manuscript. Applicable sections amended include:

***(Amended manuscript: p. 3, Lines 117 - 120, under the sub-heading 'Study context and rationale' in the 'Background' section)**:*

> The RCT allocated participants to PTSD Coach-CS or enhanced Treatment-as-Usual (e-TAU) (Bröcker et al., 2024) on a 1:1 basis. RCT participants were aged 19-61 years; female = 89%; Black = 77%, owned smartphones, could provide written informed consent, and were conversant in English. The latter was a requirement as the original version of the PTSD Coach app was.

AND

(Amended manuscript**: **p. 4, Lines 169 - 174, under the sub-heading 'Participants' in the 'Methods'):

> Of the 32 participants allocated to the PTSD Coach-CS intervention, four were lost to follow-up at post-treatment, while three participants indicated that they did not have time to complete the qualitative sub-study as they had to return to work. Thus, 25 PTSD Coach-CS intervention participants completed the 12-item questionnaire (See 'Data collection' for a more detailed description). Participants in this qualitative sub-study (aged 19-59 years; mean = 38.9; SD = 12.7) were predominantly female (80%), black (77%), and isiXhosa speaking (80%). See *Table 1* for additional socio-demographic information.

3. **p 5 line 114 maybe another word for worked-through?**

*The applicable section was amended as follows (**Amended manuscript: p. 5, Lines 179 - 181, under 'Intervention' in the 'Methods')**:*

> The intervention entailed four weekly in-person counsellor-supported sessions lasting approximately 30-40min each. At each session the registered counsellor went through and discussed selected tools of the app (See *Table 2*).

4. **Data collection: the authors should please explain the reasons for choosing a questionnaire rather than a focus group or individual interviews, as an interview is seen as a preferred method of data collection in qualitative research. This should not only be mentioned as a limitation but in the methodology section.**

*This is a valid point, and the original plan was indeed to conduct individual interviews. However, due to COVID-19 and the subsequent lockdown, this was not possible. We added the following footnote (**Amended manuscript: p. 6, Lines 194 – 195, relating to the questionnaire in the 'Methods'):***

> **Data collection**
>
> Directly after completing intervention session four, the involved counsellor invited PTSD Coach-CS participants to complete the 12-item questionnaire[4] (paper-and-pen).
>
> [3]Originally, the methodology for this qualitative sub-study included individual in-person qualitative interviews. As a more standard practice in qualitative research, this approach would have allowed for greater exploration and richer data as mentioned in the limitations of the manuscript. However, due to COVID-19 pandemic setbacks (delay in the initiation of the RCT and regulations placed in research and uncertainty at the time regarding the course and the influence of the pandemic and associated regulations on study procedures), we opted for a questionnaire in order to gather data quickly and safely.

5. **Also please discuss your thoughts on gathering information from participants that withdrew or did not complete treatment, maybe they refused, as these participants might have added valuable data.**

We acknowledge that non-completers would have added valuable feedback. However, those who did not complete the last intervention visit were lost to follow-up at post-treatment (n = 4) and, despite repeated efforts, we were not successful in contacting them.

*The following limitation was added in response to the reviewer's query (**Amended manuscript: p. 13, Lines 528 – 532 under the sub-heading 'Limitations' in the 'Conclusion')**:*

> Future studies should employ an independent team member to gather qualitative data. Lastly, while attempts were made to contact non-completers of the RCT, none were reached. It should be noted that non-completers may have provided valuable insights into improving the app and intervention as they presumably had reasons for dropping out of the study.

6. **p 6 line 154 participants should maybe be participate?**

*Thank you for raising this. The applicable section was amended as follows (**Amended manuscript: p. 6, Lines 219 – 221, under 'Methods'**):*

> **Ethical considerations**
>
> The main study (RCT) was approved by the Health Research Ethics Committee of SU (N18/03/061/ S18/05/058). During the informed consent process of the RCT, participants were informed of the voluntary nature of all study procedures (including the qualitative sub-study).

7. **p11 line 403 are you saying South Africa is working arduously towards or South Africa should work arduously towards?**

*The applicable section was amended as follows (**Amended manuscript: p. 11, Lines 486 - 490, under 'Discussion'**)*

> South Africa has a multicultural and multilingual population, and participants accessing healthcare services, and healthcare providers, face language barriers. Addressing these barriers in an effort to improve linkages to care is a priority (Hagen et al., 2020; Tate et al., 2016; Tönsing et al., 2019; van Vuuren et al., 2021).

8. **I think the authors can comment on the role of stigma and what they have learnt about stigma from their participants and how to overcome stigma in the discussion.**

Thank you for raising this. As seen in the amended background, there is a greater emphasis on treatment barriers (of which stigma is one). The role of stigma as raised by participants was reported in the 'Results' under the sub-theme originally titled 'Fear and social acceptability.' However, the title and associated information were changed to 'Stigma'. The amended sections include:

(Amended manuscript: p. 2, Lines 53 - 58, under 'Background')

> However, compared to higher income countries, LMICs typically face greater resource constraints limiting access to the appropriate care (Docrat et al., 2019; Seedat and Suliman, 2018; Singla et al., 2017). Broadly, barriers to appropriate care can be divided into (i) availability of treatments, and (ii) accessibility to available treatments, which is influenced by both systemic and personal factors (Docrat et al., 2019; Knettel et al., 2019; Sander et al., 2020).

AND

(Amended manuscript: p. 2, Lines 76 - 82, under 'Background')

> Globally and locally, recent exorbitant fuel costs and resultant increases in the cost of living (including public transport fares) hamper the ability to use public transport (Stats SA, 2021). Coupled with a high unemployment rate, the impact of rolling load shedding (i.e., rolling electricity blackouts), as well as rife and often violent public transport protests, many are unable to access PHC services (Goldberg, 2015; Laher et al., 2019; Mmakwena, 2022). Lastly, the harmful effects of stigma often prevent treatment-seeking individuals from accessing services (Booysen et al., 2021; Knettel et al., 2019; Monnapula-Mazabane and Petersen, 2021).

AND

(Amended manuscript: p. 9, Lines 350 - 352, under 'Results'):

> ***Systemic barriers to accessing treatment***
>
> Barriers to accessing treatment included travel distances and associated costs, unawareness of needing help and existing resources, stigma associated with accessing psychiatric support, and language barriers.

AND

(Amended manuscript: p. 12, Lines, 510 – 513, under 'Conclusion'):

> Utilising counsellors from the same communities as participants, who are more likely to be of similar culture and language as the patients, can also overcome cultural and language barriers in part, as well as assist with reducing stigma associated with receiving psychiatric support.

9. **The reference to "re-traumatisation" in the text also warrants some reference in the discussion. I am wondering is part of stigma not a lack of knowledge of treatment and myths around what treatment entail. The authors can perhaps say something about the role community workers or counsellors or primary healthcare clinics can play in addressing these myths. I am particularly interested in the idea of re-traumatisation and how the authors deduced from participant 8s comment that the comment refer to re-traumatisation. In no part of the manuscript do I see evidence that participants responses warrants this deduction? Can this perhaps be expanded on or is it perhaps the influence of the perceptions of the observers views of treatment. I am very aware of this idea, as it is a common theme that telling or talking about a memory of a traumatic experience (not part of this app to do any writing or telling about the trauma and therefore eliciting trauma memories?) is re-traumatising. I find this to not be a helpful word, what is rather true is that remembering can lead to the presence of emotion, thoughts, images and physical sensations as part of the memory of the trauma being activated. So it would probably be more helpful to refer to a fear of intrusion symptoms. If this is what the authors can deduct from the data.**

Thank you for raising this. As seen, the applicable sections were amended to incorporate the reviewer's thoughtful suggestions while remaining true to the data.

(Amended manuscript: p. 9, Lines 350 – 352, under 'Results')

> ***Systemic barriers to accessing treatment***
>
> Barriers to accessing treatment included travel distances and associated costs, unawareness of needing help and existing resources, stigma associated with accessing psychiatric support, and language barriers.~~, and a possible fear of re-traumatisation.~~

AND

(Amended manuscript: p. 11, Lines 422 - 428, under 'Discussion')

> Participants' dislike of detailed enquiry about the experiences of trauma was likely in reference to the monitoring sessions with the psychologist rather than the intervention or the app itself (Bröcker et al., 2024). This potential participant discomfort during PTSD-focused research is reported in the literature with caution requested when conducting such research (Brown et al., 2014; Legerski et al., 2010). However, it could also be that participants were alluding to the stigma associated with accessing psychiatric support and a lack of knowledge or misconception of what treatment entails (Booysen et al., 2021; Knettel et al., 2019; Morgan et al., 2018).

AND

(Amended manuscript: p. 11, Lines 473 - 477, under 'Discussion')

> Denial about needing support, and stigma (e.g., fear of judgement and associated concerns about the social acceptability of accessing support) were mentioned as systemic barriers to treatment (Ciccolo et al., 2021; Hundt et al., 2018; Nortjéet al., 2021; Rice et al., 2020). The

harmful effects of general stigma associated explicitly with mental illness is well-known (Booysen et al., 2021; Knettel et al., 2019; Monnapula-Mazabane and Petersen 2021).

AND

(Amended manuscript: p. 12, Lines 510 - 513, under 'Conclusion')

Utilising counsellors from the from the same communities as participants, who are more likely to be of similar culture and language as the patients, can also partly overcome cultural and language barriers, as well as assist with overcoming the stigma associated with receiving psychiatric support.

I enjoyed reading the manuscript. *Thank you.*

REVIEWER 2

Comments to the Author

Thank you for inviting me to review this article. Please find my comments to the article hereafter. I have provided detailed comments on the background and methods as I believe these need to be fundamentally changed, therefore I have not reviewed the results or discussion.

I'd recommend the authors consider following a structure such as the one used in the following article:

Puddephatt J, Leightley D, Palmer L, Jones N, Mahmoodi T, Drummond C, Rona R, Fear N, Field M, Goodwin L. A Qualitative Evaluation of the Acceptability of a Tailored Smartphone Alcohol Intervention for a Military Population: Information About Drinking for Ex-Serving Personnel (InDEx) App
JMIR Mhealth Uhealth 2019;7(5):e12267. URL: https://mhealth.jmir.org/2019/5/e12267

This should help with structuring and some of the issues I have raised in my review. You do not nor should reference this article it is merely intended as a reference.

Thank you for referring us to this informative article.

Overall key themes:

1. **Secondary analysis: From the manuscript this appears to be a secondary analysis of data collected in an RCT. This needs to be clearly stated in the manuscript. This means that the authors should focus on identifying the reasons as to why this qualitative analysis was conducted, and how it can help inform PTSD coach, not why the RCT itself was conducted.**

Please see the amended background that provides more clarity on the rationale and context of the RCT and how the qualitative sub-study comprised a secondary aim of an RCT. Now that the RCT has been published, this study can be framed in the context of the RCT (Bröcker et al., 2024).

*The amended background has a sub-heading 'Study context and rationale' under which the original RCT is described (**Amended manuscript: p. 3, Lines 112 – 140**). This section is followed by a sub-heading titled 'Present study aim' that more clearly describes why this qualitative study was conducted as part of the RCT procedures.*

*(**Amended manuscript: p. 4, Lines 142 – 150 under 'Background'**):*

> *Present study aim*
>
> One of the secondary objectives of the RCT was a qualitative sub-study to augment the findings of the RCT (Bröcker et al., 2024). To this end, we collected qualitative feedback from PTSD Coach-CS participants exploring their experiences of the feasibility (e.g., how they experienced the support received), acceptability (e.g., what they liked about the intervention), and impact (e.g., how the intervention influenced them) of PTSD Coach-CS in a South African community setting. Qualitative data provides a more in-depth understanding of participants' experiences which can be used to inform future research and intervention implementation. This manuscript presents the results of the qualitative sub-study.

2. **Referencing: Throughout the manuscript there is a lack of citations to support statements that are made. The authors should seek to include citations in the manuscript to validate and justify statements.**

Generally, references were placed at the end of sentences or paragraphs for ease of reading. However, we did note that some references were missing and added them where appropriate. For example:

*(**Amended manuscript: p. 1, Lines 52 – 53 under 'Background'**):*

> Timeous and effective interventions can mitigate the debilitating effects of psychiatric conditions like PTSD (Bisson and Olff, 2021; Seedat and Suliman, 2018).

AND

*(**Amended manuscript: p. 11, Lines 430 - 433 under 'Discussion'**):*

> Other negative comments were largely related to the desire for more human interaction and more counselling. This highlights participants' desire for direct assistance/therapy which they may not be receiving due to resource constraints (Docrat et al. 2019).

Specific comments:

3. **Abstract: From the abstract it isn't clear if the manuscript is reporting the RCT trial results, reporting a secondary aim of the Rat or a secondary analysis. It would b helpful to clarify this in the abstract. Further, the abstract makes reference to 25 participants, it isn't clear if these have been drawn from the control or intervention arm.**

*Please note, due to feedback received from the journal upon acceptance for publication of the RCT, the abstract was amended significantly to meet the strict word count (200). We have added the following: **(Amended manuscript: p. 1, Lines 2 - 6, under 'Abstract'):***

> We explored participants' experiences of a counsellor-supported PTSD Coach mobile application intervention (PTSD Coach-CS) in a randomised controlled trial. PTSD Coach-CS participants who received the intervention and, self-completed a custom-designed questionnaire at intervention completion were included were included (*n*=25; female=20; ages 19-59; isiXhosa=22).

4. **Impart statement: This should include references to source to support the statements which have been made. For example, statements related to high-levels of PTSD need to be sourced. This is important to help framing with the reader.**

This is an important comment, however, journal guidelines for the 'Impact statement' of the manuscript does not require references.

Background:

5. **It would be useful to provide context to statements such as those on line 55 to 57. For example, how does the rates of PTSD compare with other populations, such as those in the western world?**

Thank you for raising this. The amended background addresses this more fully as well as emphasising the greater lack of psychiatric support for those struggling with PTSD in LMICs (compared to higher-income countries).

(Amended manuscript: p. 1, Lines 49 – 58, under 'Background'):

> Low to middle-income countries (LMICs), such as South Africa, face high levels of psychiatric conditions including posttraumatic stress disorder (PTSD), impacting the daily functioning of those affected (Kessler et al., 2017; Ng et al., 2020; Singla et al., 2017). In sub-Saharan African countries, the PTSD prevalence is around 22% (Ng et al., 2020). Timeous and effective interventions can mitigate the debilitating effects of psychiatric conditions like PTSD (Bisson and Olff, 2021; Seedat and Suliman, 2018). However, compared to higher income countries, LMICs typically face greater resource constraints limiting access to the appropriate care (Docrat et al., 2019; Seedat and Suliman, 2018; Singla et al., 2017). Broadly, barriers to appropriate care can be divided into (i) the availability of treatments in each setting and (ii) the accessibility of treatments when available, which is influenced by both systemic and personal factors (Docrat et al., 2019; Knettel et al., 2019; Sander et al., 2020).

6. **Further, it may be useful to the reader if the authors provide a clear statement on how PTSd is measured in LMICs. For example, is it via self-report, validated questionnaire or clinical assessment?**

While the measurement of PTSD receives increasing attention in research, the measurement thereof in LMICs, etc., falls outside the scope and focus of the current article. However, to clarify which measures were used in the current dataset, please see the following:

(Amended manuscript: p. 3, Lines 121 - 127, under 'Study context and rationale' in the 'Background'):

> A registered clinical psychologist conducted the Mini-International Neuropsychiatric Interview for DSM-5 and the Clinician-Administered PTSD Scale for DSM-5 (CAPS-5) to confirm their PTSD diagnosis (Sheehan et al., 1998; Weathers et al., 2013). The psychologist, blinded to intervention allocation, monitored participants for treatment response from pre- to post-treatment at four weeks, and to one and three-month follow-up. Outcome measures included the Clinician-Administered PTSD Scale (CAPS-5) (main outcome), the PTSD Checklist (PCL-5), and the Depression, Anxiety and Stress Scale-21 items (DASS-21) self-report measures (Henry and Crawford, 2005; Wortmann et al.,2016).

7. **The authors cite that resources limitations mean that those in LMICs face barriers to assessing care. However, isn't it also the technology that is limited also? Technology adoption and technology literacy are really important factors that the authors have not discussed.**

Please see the amended background highlighting more specific barriers in LMICs, including South Africa specifically ***(Amended manuscript: p. 2, Lines 49 - 84, under 'Background').*** *Technology adoption and literacy may be both a generational barrier as well as related to income (Tomczyk et al., 2023). Results of our RCT indicated that smartphone ownership (technology adoption) and technology literacy were not significant barriers to intervention implementation noted by participants supports. Please also see the following amended section* ***(Amended manuscript: p. 3, Lines 130 - 140, under 'Study context and rationale' in 'Background'):***

> The main findings of the RCT included greater improvement in clinician-monitored PTSD and self-reported stress symptoms over time in the PTSD Coach-CS group compared to the e-TAU group (Bröcker et al., 2024). Intervention uptake was good, with most participants attending all four intervention sessions. Also, self-reported app use outside of these sessions varied from daily to five times per week (Bröcker et al., 2024). Generally, the PTSD Coach app was positively received, and participants rated the app as moderately to very helpful in managing PTSD symptoms. Smartphone ownership was not a significant barrier to intervention implementation, but technical difficulties related to app download were problematic for one participant (Bröcker et al., 2024).

8. **Line 82 to 88 describes the RCT, and not the rationale for the present study. From the background there isn't sufficient justification as to why this study is being conducted (the qualitative component).**

As mentioned in response to the first query the 'Background' section was significantly amended and addressed this more clearly.

- ***(Amended manuscript: p. 3, Lines 112 - 140, under the sub-heading 'Study context and rationale')***
- ***(Amended manuscript: p. 4, Lines 142 – 150 under the added sub-heading 'Present study aim')***

9. **Overall the background lacks any specific detail to help the reader understand the context and rationale for undertaking the study. The background needs to be extended significantly to address these concerns.**

Please see the amended 'Background' as mentioned above.

Methods:

10. **The authors should provide a copy of the CONSORT check list to help readers and reviewers match up statements to the checklist. For example, it appears that the present structure of the manuscript and title do not meet the guideline criteria.**

It is not conventional to include a CONSORT checklist and follow the related guidelines whilst writing up qualitative data. The CONSORT checklist and guidelines were used whilst writing up the RCT results (Please see Bröcker et al., 2024). However, as seen in the 'Methods' section, the COREQ was used while writing the report ***(Amended manuscript: p. 4, Lines 154 - 155, under 'Methods'):***

> We adhered to the COnsolidated criteria for REporting Qualitative research (COREQ) guidelines when writing this manuscript (Tong et al. 2007).

11. **Line 94 - can you confirm who is blinded and how?**

Please see the following ***(Amended manuscript: p. 3, Lines 121 - 125, under the subheading 'Study context and rationale' in the 'Background' section)****:*

> A registered clinical psychologist conducted the Mini-International Neuropsychiatric Interview for DSM-5 and the Clinician-Administered PTSD Scale for DSM-5 (CAPS-5) to confirm their PTSD diagnosis (Sheehan et al. 1998; Weathers et al.,2013). The psychologist, blinded to intervention allocation, assessed treatment response from pre- to post-treatment at four weeks, and to one and three-month follow-up.

12. **Line 97 age range differs from that reported in the abstract.**

This comment is likely related to initial confusion about how the qualitative dataset fits into a larger RCT study context. The amendments made to the manuscript clarify this and better distinguish between the sample characteristics of the initial RCT and those of the PTSD Coach-CS intervention arm that completed the questionnaire.

13. **Who conducted the DMS-5 interview to assess the PTSD status? Were they a researcher, a clinician or someone else?**

Please see the following ***(Amended manuscript: p. 3, Lines 121 - 125, under the subheading 'Study context and rationale' in the 'Background' section)****:*

> A registered clinical psychologist conducted the Mini-International Neuropsychiatric Interview for DSM-5 and the Clinician-Administered PTSD Scale for DSM-5 (CAPS-5) to confirm their PTSD diagnosis (Sheehan et al. 1998; Weathers et al.,2013). The psychologist, blinded to intervention allocation, monitored participants for treatment response from pre- to post-treatment at four weeks, and to one and three-month follow-up.

14. **It would be useful to provide information on what the original study was powered on. Was it change in PTSD symptoms, change in CAPS score? This needs to be stated.**

Please see (Bröcker et al., 2024), as this information is better suited to the RCT-focused paper.

15. **How were the participants sampled? Were they invited to take part alongside the main RCT, or after they'd taken part? Linked in with this, were the interviews conducted online, or in person?**

Thank you for raising this. The first part of this query is addressed in the above responses with adding more context to the initial RCT in the current manuscript. Additionally, please see the following ***(Amended manuscript: p. 5, Lines 179 - 180, under 'Methods')****:*

> **Intervention**
> The intervention entailed four weekly in-person counsellor-supported sessions lasting approximately 30-40min each.

AND

(Amended manuscript: p.5, Lines 194 - 195, under 'Methods')*:*

> **Data collection**
> Directly after completing intervention session four, the counsellor invited PTSD Coach-CS participants to complete the 12-item questionnaire (paper-and-pen).

16. **Do the authors have access to any process evaluation measures collected in the study? Such as app analytics, interactions and time spent?**

To avoid significant self-plagiarism, we summarised information regarding the initial RCT in this manuscript where we also reference the RCT paper. Other information (including information related to this query) is reported in the RCT paper (Please see: Bröcker et al., 2024). Not using a research version of the app is also noted as a limitation in the paper.

17. **Further, why wasn't a usability questionnaire used such as the mHealth App Usability Questionnaire (MAUQ)?**

Thank you for recommending this questionnaire, which we noted for future research planning. As this was a sub-study of a larger RCT, the main focus was on RCT quantitative-related measures to caution against participant burnout. However, this suggestion was incorporated in the amended manuscript ***(Amended manuscript: p.12, Lines 525 - 526, under the sub-heading 'Limitations' in the 'Conclusion'):***

> Future research should use verbal interviews to allow for clarification and richer data. Relatedly, adding a usability questionnaire, such as the mHealth App Usability Questionnaire, can further enrich the data.

REVIEWER 3

Comments to the Author

Overall, this is a well-written paper that provides useful qualitative data on an evaluation of a counsellor supported application of PTSD-Coach. Overall, I would suggest that the contribution of this manuscript is not just in its findings, but rather also in its methodological approach. As such, I would suggest that the most important revisions/additions at this phase might be to provide more detail on the approach. That is, how were counselors trained? What specifically was the flow/structure of each section? How were counselors assessed for fidelity/implementation quality? Since this is the first counsellor-assisted application of PTSD coach in a resource constrained context, these details would be useful for both researchers (for replication purposes) and for practitioners (to understand implementation guidance).

Thank you for the comments and queries. We acknowledge that this article needed more context and is now better suited for publication since the publication of the initial RCT results (Please see: Bröcker et al., 2024). There is now sufficient published information for replication purposes as well. The 'Background' section of this qualitative manuscript was significantly amended, including referencing the RCT manuscript ***(Amended manuscript: p. 3, Lines 137 – 140 under 'Study context and rationale'):***

> Overall, the RCT results supported the feasibility and acceptability of both the original version of the PTSD Coach mobile app platform and the notion of involving counsellors in intervention delivery (Bröcker et al., 2024). For more information about the original RCT (e.g., counsellor training and protocol fidelity), please see (Bröcker et al., 2024).

While we were mindful of not duplicating too much of the RCT manuscript information, as well as of journal article word count requirements, more information can be added should the reviewers think it necessary.

1. **It would be helpful, in the results section of the abstract, to cite a couple of the primary concerns and barriers participants raised about the intervention.**

Thank you for raising this. Please note, due to feedback received from the journal upon acceptance for publication of the RCT data, the abstract was amended significantly to meet the strict word count (200). In response to this query specifically, please see the following ***(Amended manuscript: p. 1, Lines 9 - 15, under 'Abstract')****:*

> Three main themes emerged. (i) Participants' largely positive experiences of treatment procedures included the safe space created by the counsellor-support in combination with the PTSD Coach application; allowing them to learn about and understand their lived experiences, and to accept their PTSD diagnoses. (ii) Positive perceptions of the PTSD Coach application, yet raising important concerns (e.g., lack of family involvement) for future consideration; and (iii) Intervention-specific and systemic treatment barriers (e.g., stigma) providing important information to inform and increase the usefulness of the PTSD Coach-CS intervention.

2. **The phrasing of the first sentences in the impact statement/intro need to be re-worked as the phrasing makes it sound as if countries have PTSD rather than LMICs facing high rates of PTSD prevalence in their population. A similar issue is present in the third sentence of the intro related to access to care.**

The applicable sections were amended as follows:

(Amended manuscript: p. 1, Lines 23 – 31, under 'Impact Statement')

> Low and middle-income countries (LMICs), such as South Africa, often face healthcare barriers with many individuals not accessing needed support due to resource constraints. To identify feasible and effective intervention alternatives for trauma survivors in LMICs, we evaluated a counsellor-supported PTSD Coach mobile application (PTSD Coach-CS) intervention in a randomised controlled trial in a South African adult community sample. As part of the evaluation, we explored participants' experiences of the intervention to inform and complement the findings of the RCT.

(Amended manuscript: p. 2, Lines 49 – 58, under 'Background')

> Low to middle-income countries (LMICs), such as South Africa, face high levels of psychiatric conditions including posttraumatic stress disorder (PTSD), impacting the daily functioning of those affected (Kessler et al., 2017; Ng et al., 2020; Singla et al., 2017). In sub-Saharan African countries, the PTSD prevalence is around 22% (Ng et al., 2020). Timeous and effective interventions can mitigate the debilitating effects of psychiatric conditions like PTSD (Bisson and Olff, 2021; Seedat and Suliman, 2018). However, compared to higher-income countries, LMICs typically face greater resource constraints limiting access to the appropriate care (Docrat et al., 2019; Seedat and Suliman, 2018; Singla et al., 2017). Broadly, barriers to appropriate care can be divided into (i) availability of treatments and (ii) accessibility to

available treatments, which is influenced by both systemic and personal factors (Docrat et al., 2019; Knettel et al., 2019; Sander et al., 2020).

3. **It would be useful in the first paragraph of the introduction, to provide some information about PTSD prevalence and mental health care access in South Africa, specifically.**

Please see the applicable amended sections in the 'Background' in response to the query above. The introduction has more emphasis on the greater lack of psychiatric support and greater need for innovative treatment alternatives (and not PTSD prevalence per se) for those struggling with PTSD in LMICs (compared to higher-income countries).

4. **It is surprising that no adaptations to the PTSD Coach app – in either substance or language were made. Can the authors speak a bit to the rationale for this? Was the suitability of the application as originally developed for the US DoD evaluated in any formal way for use in this context? This is especially important to address since the participants listed English-only services and a perception that services are "too western" as barriers to care. I was glad to see this raised in the discussion section, but I think the rationale for testing it this way should be presented earlier in the paper.**

Thank you for raising this. As the reviewer may be aware, app development and adaptation are costly. Hence, we opted to first trial and evaluate the original version of the app, including qualitative feedback from the users (participants). Thus, the larger RCT and the qualitative sub-study was our way of evaluating the suitability of the app in our setting. To address these comments, the following footnote was added.

(Amended manuscript: p. 3, Lines 113 - 121, under 'Background'):

Study context and rationale

To address the aforementioned gap in the extant literature on PTSD Coach, we conducted a randomised controlled trial (RCT) to evaluate the effectiveness of a brief four-session counsellor-supported PTSD Coach mobile app (PTSD Coach-CS) intervention in South African adults with PTSD (trial registration number: PACTR202108755066871) (Bröcker et al., 2024). Study flyers were distributed in the community and promoted on social media. The RCT allocated participants to PTSD Coach-CS or enhanced Treatment-as-Usual (e-TAU) (Bröcker et al., 2024) on a 1:1 basis. RCT participants were aged 19-61 years; female = 89%; Black = 77%, owned smartphones, could provide written informed consent, and were conversant in English. The latter was a requirement as the original version of the PTSD Coach app was only available in English.[1]

[1]Due to cost constraints, the original version of the app was not adapted before use in the RCT. However, first conducting an RCT and subsequent qualitative sub-study allowed us to gain insight into whether and what adaptation is needed; thereby preventing unnecessary costs for adaptation.

In the amended manuscript, where we present more RCT findings in the 'Background', we also comment on the feasibility and acceptability of the original version of the app in our setting ***(Amended manuscript: p.1, Lines 132 – 139, under the subheading 'Study context and rationale' in the 'Background'):***

Intervention uptake was good, with most participants attending all four intervention sessions, and self-reported app use outside of these sessions varied from daily to five times per week (Bröcker et al., 2024). Generally, the PTSD Coach app was positively received, and participants rated the app as moderately to very helpful in managing their PTSD symptoms. Smartphone ownership was not a significant barrier to intervention implementation, but technical difficulties related to app download was problematic for one participant (Bröcker et al., 2024). Overall, the RCT results supported the feasibility and acceptability of both the original version of the PTSD Coach mobile app platform and the notion of involving counsellors in intervention delivery (Bröcker et al., 2024).

*The above links to what is written in the 'Conclusion' of the manuscript (**Amended manuscript: 12, Lines 507 - 515):***

> Notably, the data provided insight into the barriers and what needs to be improved on in terms of intervention implementation. For instance, training registered counsellors, preferably from the respective communities, to provide the PTSD Coach-CS intervention at local clinics under supervision limits travel and associated costs. Utilising counsellors from the same communities as participants, who are more likely to be of similar culture and language as the patients, can also overcome cultural and language barriers in part, as well as assist with reducing stigma associated with receiving psychiatric support. Adapting the PTSD Coach app culturally and language-wise (e.g., isiXhosa or Afrikaans) can further assist in greater intervention adoption and usefulness, however, the counsellor support might be a better use of resources and of value to PTSD sufferers.

Since the use of the original app version was not a significant barrier, we did not expand on this in the 'Background,' however, should the reviewer think that more than the footnote should be added, this can be further addressed.

5. **Please add more information about counselor training and experience. Were counsellors restricted from providing support outside of education/co-use of the app? If so, how was fidelity assessed? Were counselors assessed in any way in terms of their ability to correctly respond to participant questions?**

*Please see the following **(Amended manuscript: p. 3, Lines 137 - 140, under 'Study rationale and context in the 'Background'):***

> Overall, the RCT results supported the feasibility and acceptability of both the original version of the PTSD Coach mobile app platform and the notion of involving counsellors in intervention delivery (Bröcker et al., 2024). For more information on the original RCT (e.g., counsellor training and protocol fidelity), please see Bröcker and colleagues 2024.

AND

(Amended manuscript: p. 5, Lines 181 - 182, under 'Intervention' in the 'Methods'):

> The counsellor provided a standard method of support (e.g., assisting with language and technology difficulties without providing therapeutic input) as per intervention protocol.

More detail on the rationale for restricting the therapeutic involvement of the counsellor can be found in the RCT paper. However, should the reviewer think more should be added to this manuscript as well, this can be addressed.

6. **Was participant app use outside of sessions tracked in any way?**

As mentioned, this information is more suited to the RCT manuscript, and we were cautious not to duplicate information. (Please see Bröcker et al., 2024).

7. **Please provide the exact questions asked of participants in the methods section or as an electronic supplement.**

*Apologies, while this was submitted as supplemental material (Appendix A: Questionnaire), it appears the explicit mention thereof was omitted. Please see the applicable section amended as follows (**Amended manuscript: p. 6, Lines 194 – 197, under 'Methods'):***

> **Data collection**
>
> Directly after completing intervention session four, the counsellor invited PTSD Coach-CS participants to complete the 12-item questionnaire (paper-and-pen).[1] This self-administered questionnaire elicited data on the feasibility, acceptability, and the potential impact of the PTSD Coach-CS intervention (see Appendix A: Questionnaire for further details).

8. **What was the interrater reliability of the two coders?**

While we are aware that qualitative research can entail interrater reliability, we did not undertake interrater reliability. This is not a requirement for thematic analysis, according to Braun and Clarke (2014).

9. **What were the monitoring sessions with the psychologist? This is referenced for the first time in the results, but is not detailed in the methods. Does this refer to the pre/post assessments?**

*Thank you for raising this. Please see the following (**Amended manuscript: p. 3 Lines 121 - 125, under the sub-heading 'Study context and rationale' in the 'Background'**):*

> A registered clinical psychologist conducted the Mini-International Neuropsychiatric Interview for DSM-5 and the Clinician-Administered PTSD Scale for DSM-5 (CAPS-5) to confirm their PTSD diagnosis (Sheehan et al., 1998; Weathers et al.,2013). The psychologist, blinded to intervention allocation, assessed treatment response from pre- to post-treatment at four weeks, and to one and three-month follow-up.

10. **Theme saturation within the sample is unclear – it would be useful to document how many participants' responses were coded under each theme, and note any interrelations between themes (e.g., did participants have a balanced view of the app with some negative and some positive comments or did participants typically express either positive or negative views – this would have different implications for care).**

Due to the nature of data collection (questionnaire responses), all responses were analysed. For the benefit of the reviewer, we include a table with frequency distributions calculated from the dataset. This corresponds with the results (e.g., when we refer to 'most participants were positive about the intervention'), however we did not include this in the manuscript per se.

**How did you feel about the process?**	***n* (%)**
I liked it.	14 (56%)
I liked it, but… (e.g., does not involve family)	5 (20%)
**Was the app helpful?**	
Yes.	17 (68%)
Yes, to some degree… (e.g., wanted more counselling)	7 (28%)
No (e.g., liked talking to counsellor/psychologist)	1 (4%)
**Can others benefit from the app?**	***n* (%)**
Yes.	14 (56%)
Yes, but… (e.g., not aware of the app)	9 (36%)

Note. Please note that some participants did not answer all the questions or responded with answers unrelated to the question. Thus, the frequencies do not necessarily add to 100%.

Relatedly, a column was added (Appendix A: Questionnaire) to reflect the number of responses analysed.

**12-item questionnaire**	**Number of responses**
1. What did you like/not like about the process?	**25**
2. What was helpful/unhelpful?	**25**
3. What do you think about PTSD Coach?	**25**
4. How could others benefit from it?	**24**
5. How did you come to participate in this study?	**24**
6. What do you think are the barriers to accessing treatment?	**23**
7. What do you think prevents others from receiving treatment?	**23**
8. What does your community think about counselling/receiving help?	**21**
9. How did you experience the support received from the volunteer counsellor?	**21**
10. How has the intervention influenced you?	**20**
11. Did Covid-19 have an impact (positively or negatively) on your use of the app?	**18**
12. Any other comments, thoughts, or suggestions?	**20**

11. **Spelling error ("therefor" on page 9, line 08)**

*Thank you for raising this. The following was amended (**Amended manuscript: p. 10, Lines 387, under 'Systemic barriers to accessing treatment' in the 'Results.')**:*

> *Support*
>
> Participants' responses indicated a perceived lack of resources as contributing to treatment barriers, noting *"Availability of counsellors,"* (P4) and that *"The clinics in SA does not provide the support people want - Therefore people have nowhere to go"* (P11).

12. **The finding re: lack of family involvement and reflects a broader need in LMIC/conflict-affected settings (e.g., Jordans, et al., 2019).**

Yes, it does indeed and should be addressed in future research (e.g., evaluating the PTSD Coach Family app in a South African resource-constrained setting).

13. **Is there any information yet available from the larger trial on the effectiveness of this intervention vs. EUC? If so, it would be helpful to mention/cite and to discuss in light of the qualitative findings.**

As seen, the RCT results are available, were incorporated and referenced in the amended manuscript, and assist with reporting and understanding of the qualitative results.
