# Supplementary material for: Participants’ experiences of a counsellor-supported PTSD Coach intervention in a resource-constrained setting
Source: Glob Ment Health (Camb). 2024 Mar 11;11:e36. doi: 10.1017/gmh.2024.34 (PMC10988172; doi:10.1017/gmh.2024.34)
Supplement: Bröcker et al. supplementary material 3 — Bröcker et al. supplementary material [file S2054425124000347sup003.docx]

*Appendix A: Questionnaire and response frequencies*

| **12-item questionnaire** | **Number of responses analysed** |
| --- | --- |
| 1. What did you like/not like about the process? 2. What was helpful/unhelpful? 3. What do you think about PTSD Coach? 4. How could others benefit from it? 5. How did you come to participate in this study? 6. What do you think are the barriers to accessing treatment? 7. What do you think prevents others from receiving treatment? 8. What does your community think about counselling/receiving help? 9. How did you experience the support received from the volunteer counsellor? 10. How has the intervention influenced you? 11. Did Covid-19 have an impact (positively or negatively) on your use of the app? 12. Any other comments, thoughts, or suggestions? | **25**  **25**  **25**  **24**  **24**  **23**  **23**  **21**  **21**  **20**  **18**  **20** |
