# Supplementary material for: Participants’ experiences of a counsellor-supported PTSD Coach intervention in a resource-constrained setting
Source: Glob Ment Health (Camb). 2024 Mar 11;11:e36. doi: 10.1017/gmh.2024.34 (PMC10988172; doi:10.1017/gmh.2024.34)
Supplement: Bröcker et al. supplementary material 4 — Bröcker et al. supplementary material [file S2054425124000347sup004.pdf]

**We responded to the below questions based on our study which collected qualitative data via a self-completed questionnaire. We were unable to find a reporting checklist for this purpose specifically. We amended responses accordingly.*

Consolidated criteria for reporting qualitative studies (COREQ): 32-item checklist

Developed from:
Tong A, Sainsbury P, Craig J. Consolidated criteria for reporting qualitative research (COREQ): a 32-item checklist for interviews and focus groups. *International Journal for Quality in Health Care*. 2007. Volume 19, Number 6: pp. 349 – 357

YOU MUST PROVIDE A RESPONSE FOR ALL ITEMS. ENTER N/A IF NOT APPLICABLE

No. Item	Guide questions/description	Reported on Page #
**Domain 1: Research team and reflexivity**	**Data collected via self-completed questionnaires*	
Personal Characteristics		
1. Inter viewer/facilitator	Which author/s conducted the inter view or focus group?	*Self completed questionnaires. See pg 4.*
2. Credentials	What were the researcher's credentials? E.g. PhD, MD	Research team responsible for analysis: See Data analysis, pg.5
3. Occupation	What was their occupation at the time of the study?	Research team responsible for analysis: See Data analysis, pg.5
4. Gender	Was the researcher male or female?	
5. Experience and training	What experience or training did the researcher have?	Research team responsible for analysis: See Data analysis, pg.5
Relationship with participants		
6. Relationship established	Was a relationship established prior to study commencement?	N/A
7. Participant knowledge of the interviewer	What did the participants know about the researcher? e.g. personal goals, reasons for doing the research	N/A
8. Interviewer characteristics	What characteristics were reported about the inter viewer/facilitator? e.g. Bias, assumptions, reasons and interests in the research topic	N/A
**Domain 2: study design**		
Theoretical framework		
9. Methodological orientation and Theory	What methodological orientation was stated to underpin the study? e.g. grounded theory, discourse analysis, ethnography, phenomenology, content analysis	See Data analysis, pg.7
Participant selection		
10. Sampling	How were participants selected? e.g. purposive, convenience, consecutive, snowball	See Settings and participants, pg.2. See data collection, pg.4.

11. Method of approach	How were participants approached? e.g. face-to-face, telephone, mail, email	See Setting and participants, pg.2<br>See data collection, pg.4
12. Sample size	How many participants were in the study?	See setting and participants,pg.2
13. Non-participation	How many people refused to participate or dropped out? Reasons?	See Setting and participants, pg.2
Setting		
14. Setting of data collection	Where was the data collected? e.g. home, clinic, workplace	See Setting and participants, pg.2. See data collection, pg.4
15. Presence of non-participants	Was anyone else present besides the participants and researchers?	N/A
16. Description of sample	What are the important characteristics of the sample? e.g. demographic data, date	See Setting and participants, pg.2.
Data collection		
17. Interview guide	Were questions, prompts, guides provided by the authors? Was it pilot tested?	Custom designed questionnaire. Described under data collection, pg.4 Also see Appendix A
18. Repeat interviews	Were repeat inter views carried out? If yes, how many?	N/A
19. Audio/visual recording	Did the research use audio or visual recording to collect the data?	N/A
20. Field notes	Were field notes made during and/or after the inter view or focus group?	N/A
21. Duration	What was the duration of the inter views or focus group?	N/A
22. Data saturation	Was data saturation discussed?	See Data analysis, pg.5
23. Transcripts returned	Were transcripts returned to participants for comment and/or correction?	N/A. See pg.11 Limitations about our data collection method.
**Domain 3: analysis and findings**		
Data analysis		
24. Number of data coders	How many data coders coded the data?	2, See Data analysis, pg.5
25. Description of the coding tree	Did authors provide a description of the coding tree?	See Results pg, 5 Figure 1
26. Derivation of themes	Were themes identified in advance or derived from the data?	Derived from data, See Data anlysis, pg.7
27. Software	What software, if applicable, was used to manage the data?	Excel, Atals.ti.<br>See Data collection, pg.4 and Data analysis, pg.5.
28. Participant checking	Did participants provide feedback on the findings?	See limitations, pg.11
Reporting		
29. Quotations presented	Were participant quotations presented to illustrate the themes/findings? Was each quotation identified? e.g. participant number	Yes. See results pgs. 5-9
30. Data and findings consistent	Was there consistency between the data presented and the findings?	Yes. See results pgs. 5 to 9; Discussion pgs.9 to 11.
31. Clarity of major themes	Were major themes clearly presented in the findings?	Yes. See results pgs 5 to 9 and Figure 1
32. Clarity of minor themes	Is there a description of diverse cases or discussion of minor themes?	Yes. See results pgs. 5 to 9

Once you have completed this checklist, please save a copy and upload it as part of your submission. When requested to do so as part of the upload process, please select the file type: *Checklist*. You will NOT be able to proceed with submission unless the checklist has been uploaded. Please DO NOT include this checklist as part of the main manuscript document. It must be uploaded as a separate file.
