# Supplementary material for: Participants’ experiences of a counsellor-supported PTSD Coach intervention in a resource-constrained setting
Source: Glob Ment Health (Camb). 2024 Mar 11;11:e36. doi: 10.1017/gmh.2024.34 (PMC10988172; doi:10.1017/gmh.2024.34)
Supplement: Peer review history

# Editor report: Recommendation: Participants’ experiences of a counsellor-supported PTSD Coach intervention in a resource-constrained setting — R0/PR2

**DOI:** 10.1017/gmh.2024.34.pr2

**Affiliations:** Johns Hopkins University Bloomberg School of Public Health, United States

No accompanying comment.

# Editor report: Decision: Participants’ experiences of a counsellor-supported PTSD Coach intervention in a resource-constrained setting — R0/PR3

**DOI:** 10.1017/gmh.2024.34.pr3

**Affiliations:** Johns Hopkins University Bloomberg School of Public Health, United States

No accompanying comment.

# Editor report: Recommendation: Participants’ experiences of a counsellor-supported PTSD Coach intervention in a resource-constrained setting — R1/PR5

**DOI:** 10.1017/gmh.2024.34.pr5

**Affiliations:** Johns Hopkins University Bloomberg School of Public Health, United States

No accompanying comment.

# Editor report: Decision: Participants’ experiences of a counsellor-supported PTSD Coach intervention in a resource-constrained setting — R1/PR6

**DOI:** 10.1017/gmh.2024.34.pr6

**Affiliations:** Johns Hopkins University Bloomberg School of Public Health, United States

No accompanying comment.

# Editor report: Recommendation: Participants’ experiences of a counsellor-supported PTSD Coach intervention in a resource-constrained setting — R2/PR8

**DOI:** 10.1017/gmh.2024.34.pr8

**Affiliations:** Johns Hopkins University Bloomberg School of Public Health, United States

No accompanying comment.

# Editor report: Decision: Participants’ experiences of a counsellor-supported PTSD Coach intervention in a resource-constrained setting — R2/PR9

**DOI:** 10.1017/gmh.2024.34.pr9

**Affiliations:** Johns Hopkins University Bloomberg School of Public Health, United States

No accompanying comment.
